# Emergence and development of gut motility in the chicken embryo

**DOI:** 10.1371/journal.pone.0172511

**Published:** 2017-02-21

**Authors:** N. R. Chevalier, V. Fleury, S. Dufour, V. Proux-Gillardeaux, A. Asnacios

**Affiliations:** 1 Laboratoire Matière et Systèmes Complexes, Université Paris Diderot/CNRS UMR 7057, Sorbonne Paris Cité, Paris, France; 2 INSERM, U955, Equipe 6, France & Université Paris Est, Faculté de médecine, Créteil, France; 3 Membrane Traffic in Health and Disease Lab, Institut Jacques Monod, UMR 7592, CNRS & Univ. Denis Diderot, INSERM U950, Paris; Laboratoire de Biologie du Développement de Villefranche-sur-Mer, FRANCE

## Abstract

The gastrointestinal tract transports the food bolus by peristalsis. Gut motility starts at an early age in the developing embryo, well before it is required for nutrition of the organism. We present a comprehensive kinematic study of the emergence and physiological development of gut motility in all regions of the lower digestive tract of the chicken embryo from embryonic days E5 through E9. We characterized motility emergence time, propagation patterns, speed, frequency and amplitude of peristalsis waves. We found that the emergence of an uninterrupted circular ring of smooth muscle correlated with the appearance of propagative contractile waves, at E6 in the hindgut and midgut, and at E9 in the caecal appendix. We show that peristalsis at these stages is critically dependent on calcium and is not mediated by neurons as gut motility is insensitive to tetrodotoxin and takes place in the hindgut in the absence of neurons. We further demonstrate that motility also matures in *ex-vivo* organ culture. We compare our results to existing literature on zebrafish, mouse and human motility development, and discuss their chronological relationship with other major developmental events occurring in the chicken embryonic gut at these stages. Our work sets a baseline for further investigations of motility development in this important animal model.

## Introduction

Peristalsis is one of the most fundamental and widespread active transport modes of fluids to have been evolved by multicellular organisms to overcome the limitations of diffusion. Peristaltic transport is achieved by the wave-like propagation of radial and longitudinal constrictions. Among human organs, the gut is best known to perform peristalsis to propel the food bolus; the uterus and uterine ducts uses peristalsis to accelerate sperm transport to the egg cell [1]; urine is propelled through the ureter by means of peristalsis [2]. In other amniotes, blood transport has been shown to be assisted by peristalsis to overcome hydrodynamic load losses, such as in the mesenteric venous return of the chicken embryo [3] or in bat wings [4]. Peristalsis has been evolved in very distant phyla: the hydra, a member of the cnidarians, a family of non-bilaterian ocean-dwelling animals, has been shown [5] to perform peristaltic movements much akin to those of the esophagus and of the intestine. Even organisms that do not have muscle rely on a form of peristalsis [6] to transport nutrients, like the non-metazoan mold *Physarum Polycephallum*, which forms a network of tubes that can constrict using actin and myosin. The ingredients required to develop peristaltic transport are: 1°) excitable contractile elements, such as actomyosin or muscles, 2°) coupling of the contractile elements, via gap junctions for example. Since these requirements are part of the basic building blocks of most multi-cellular organisms, it is not surprising that peristalsis has appeared in such a wide range of animals.

In amniotes, peristalsis-like motion of the gut (hereafter referred to as “motility”) first appears in the embryo while it is still being supplied with nutrients via the placenta (or yolk sac), i.e., well before this motion is actually required for nutrition. The ontogenesis of gut motility has been the object of recent research: Roberts et al. [7] have detected the first contractile waves at E13.5 in the mouse gastrointestinal (GI) tract and they have shown that this early motion is purely myogenic—it does not require the participation of interstitial cells of Cajal (ICC) or of neurons. Studying gut motility in the zebrafish embryo, Holmberg et al. [8] came to similar conclusions: gut movement at 4 days post fertilization (dpf) is purely myogenic, neurons only come into play later. Does this early motility play any physiological role for the developing gut? Are these conspicuous, energy-consuming contractions a necessary learning process [9] the embryo has to go through towards a vital, functional motility at birth/hatching? If so, do these early movements provide a basic framework for future wiring of the ICC network and of the enteric nervous system (ENS)? Or is early myogenic motility a remnant of a more primitive form of peristalsis?

In an effort to start addressing these questions, we investigate here how gut motility comes about in the chicken embryo. Because of its widespread availability, relatively quick development and ease-of-access, the chicken embryo has historically been one of the most widely used models for vertebrate developmental studies [10]. While recent contributions have outlined with great precision the morphological changes and cell differentiation events taking place within the avian gut tract [11], no study has to our knowledge examined the emergence and development of dynamics (= time-variable smooth muscle contractions) within this organ. The goal of the present study is therefore to provide a detailed description of the dynamics taking place in the gut of the chicken embryo from E5 to E9. We further show that motility in this developmental period is independent of neural activity but relies on calcium fluxes. We finally demonstrate that peristalsis also develops in *ex-vivo* organ culture, thus providing a method to study the biochemical and physical inputs required for the development of this physiological reflex.

## Results

### Motility analysis

**Fig 1:**
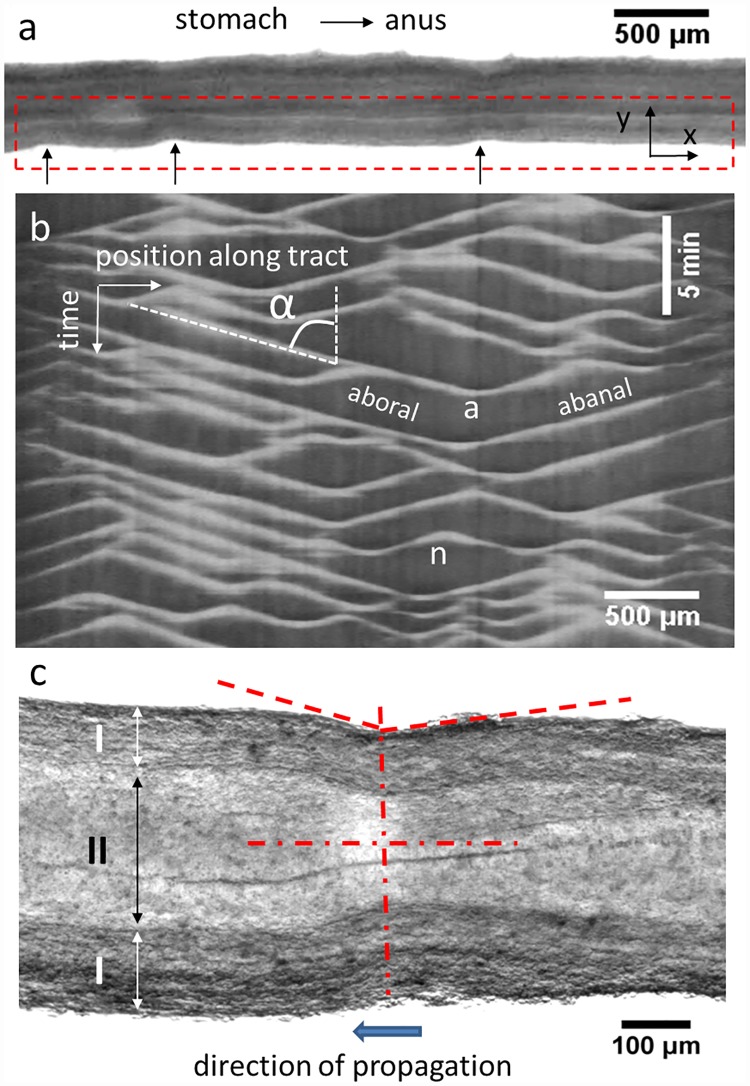
Analysis of embryonic gut motility. a) Stereomicroscope single frame from a time-lapse stack showing an E9 jejunum resting on an Anodisc membrane. Black arrows: three constrictions travelling from right to left (abanal waves). b) Motiligram (= kymograph) computed from the region-of-interest boxed by red dashed lines in a). Aborally and abanally propagating waves are indicated, as well as wave annihilation (“a”) and nucleation events (“n”); the speed *v* of a wave is deduced from the tilt angle *α* by *v* = tan *α*. c) Magnified view of a constriction (E8 jejunum). The constriction is not symmetric (dashed red lines): the edge of the gut ahead of the propagating constriction has a steeper incline than the edge of the gut in back of the constriction. I: smooth muscle and myenteric plexus region; II: mucosa, epithelium and lumen region. The deformation (= strain) due to the constriction is concentrated in region II; the thickness of region I does not change during the constriction. The full time-lapse video is available as S1 Video.

Embryonic chick gut dynamics consisted of radial constrictions of the gut wall that propagated along the GI tract in either aboral (anterior to posterior) or abanal (posterior to anterior) directions (S1 Video). We characterized these dynamics by tracing a region-of-interest (ROI) around one edge of the gut and performing the “reslice” operation with the ImageJ software. “Reslice” computes for each image in the stack the Y-averaged pixel intensity along the X-axis of the ROI, thus reducing each image to a horizontal line of variable pixel intensity. The constrictions (black arrows in Fig 1a) cause a local change of pixel intensity.

**Fig 2:**
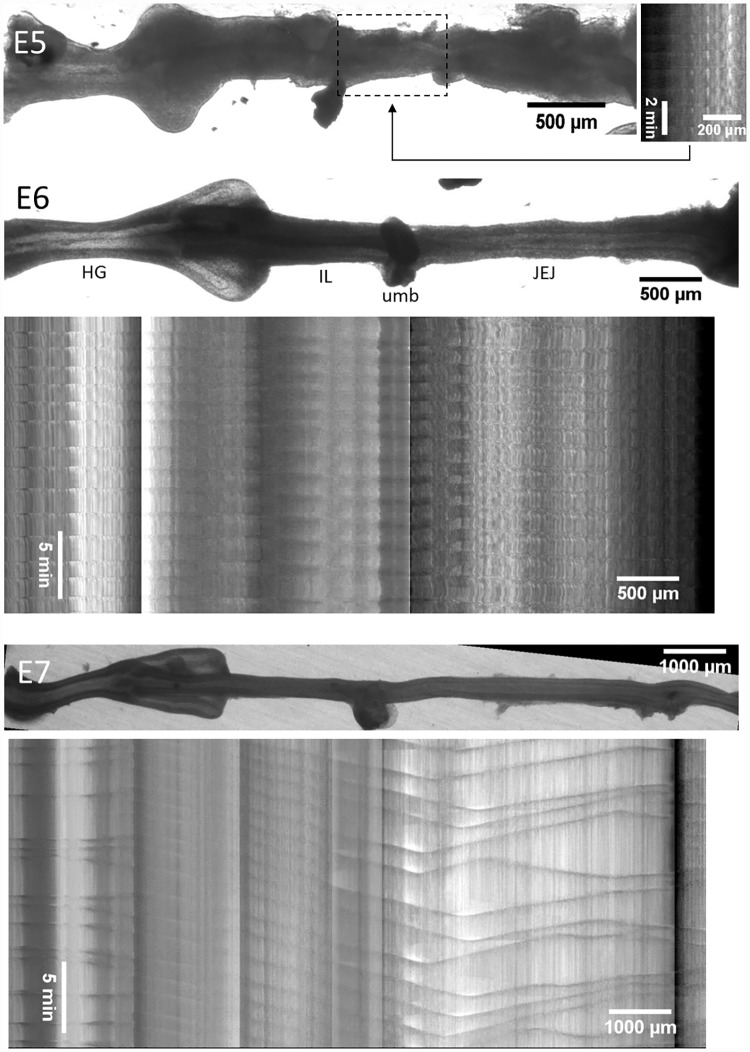
Representative motility patterns at E5, E6 and E7 (S2–S4 Videos). At E5, the motiligram is deduced from the sole beating region of the gut which is the distal jejunum (dashed box). At later stages, the motiligram is placed right below the region of the gut from which it was derived; spatial scales are the same for still images of the gut and motiligrams. The main anatomical segments of the gut are indicated at E6, HG: hindgut, IL: ileum, JEJ: jejunum, umb: umbilicus.

**Fig 3:**
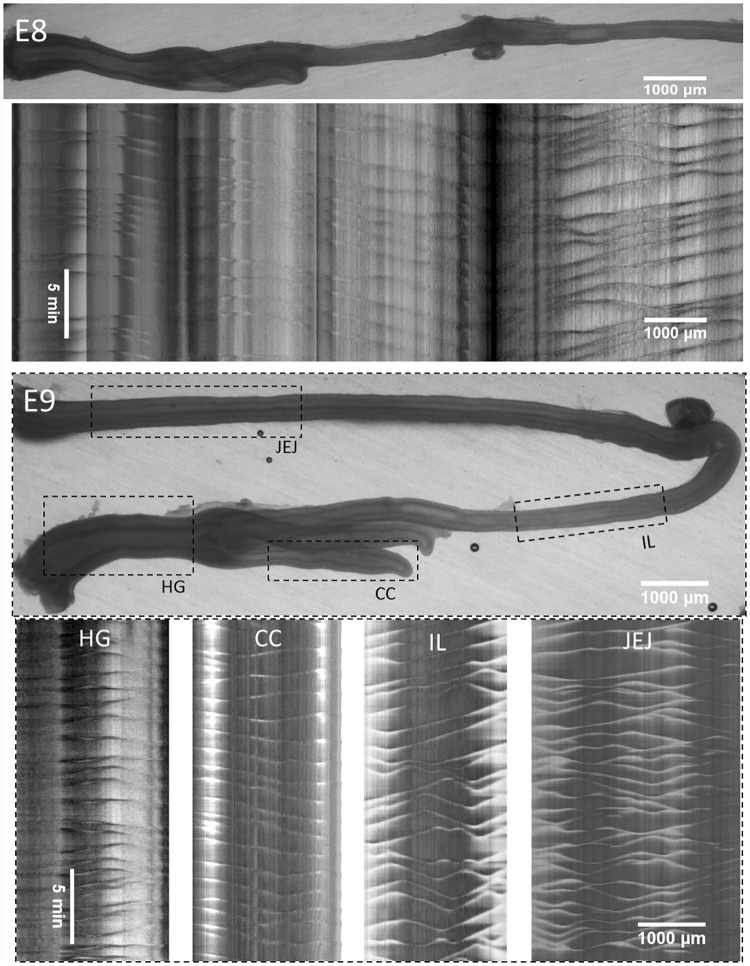
Representative motility patterns at E8 and E9 (S5 and S6 Videos). The E8 motiligram is placed right below the region of the gut from which it was derived; at E9, separate motiligrams were derived from regions indicated with dashed boxes. HG: hindgut, IL: ileum, JEJ: jejunum, umb: umbilicus, CC: caecal appendix.

The different lines from the different frames in the stack are then laid side-by-side along the vertical axis, resulting in a two-dimensional image in which the x-axis is the spatial x-coordinate along the long-axis of the gut segment being analyzed, and the y-axis is time (frames of the movie). Such spatiotemporal diagrams are commonly called “kymographs” (greek for “wave writer”); in this report we introduce the more specific and suggestive term “motiligram”. A typical motiligram is shown in Fig 1b. Each propagating constriction wave gives rise to a slanted, straight, white line. Descending and ascending lines are respectively associated to aborally and abanally propagating waves. The speed of the wave is related to the tilt angle *α* of each wave by *v* = tan *α*. We found that aboral and abanal waves propagated at the same speed. When two waves propagating in opposite directions met, they annihilated each other (V-shaped endings, indicated by an “a” in Fig 1b). Conversely, Λ-shapes corresponded to the nucleation of two oppositely-propagating waves (indicated by an “n” in Fig 1b). We characterized motility by computing the speed of the waves and their frequency *f* = *n*/Δ*t*, where *n* is the number of waves passing through a given position in a time Δ*t*. The amplitude was measured directly from the video as *A* = (*d*_*rest*_ − *d*_*constricted*_)/*d*_*rest*_, where *d*_*rest*_ is the resting diameter of a point along the gut and *d*_*constricted*_ is the diameter at the same position when it is constricted by a peristalsis wave. Unlike in the work of Roberts et al. [7], the intensity of the white lines in the motiligrams shown here is not directly proportional to the amplitude of the waves. In particular, in addition to the reduction in diameter, which leads to an increase in pixel intensity, the constriction also leads to a local decrease in the light transmitted through the gut and thus to a decrease of the mean intensity per pixel. For relatively shallow waves (E6-E8), the latter is the dominating effect, so that the contrast is inverted and propagating waves appear as dark lines on a bright background (see E6-E8 motiligrams, Figs 2 & 3). We measured the frequency, speed and amplitude in the hindgut, caeca, ileum and jejunum; frequency and amplitude depended only weakly on the choice of a particular point along the gut segment as long as the number of waves over which the measurement was performed was high enough (~15).

Fig 1c shows a cell-resolved magnification of a constriction at E8. The direction of propagation of the wave can be deduced from a still image because the slope of the gut edge ahead of the constriction is steeper than that in back of the constriction. This is due to the fact that it takes some time for the tissue to come back to its initial shape after it has been “pinched” by the propagating constriction. Fig 1c also reveals that the tissue layer that is compressed by the contractile wave is the epithelium, mucosa and lumen (region II); the thickness of the muscle and myenteric plexus region (region I) remains constant during contraction. This means that the physical strain in the mucosa and epithelium *s* = (*d*_*rest*,*II*_ − *d*_*constricted*,*II*_)/*d*_*rest*,*II*_ (where the resting and constricted diameters of only region II are considered) is greater than the amplitude defined on the basis of changes in the external diameter, *A* = (*d*_*rest*_ − *d*_*constricted*_)/*d*_*rest*_. For instance, in the case of the constriction shown in Fig 1c, the amplitude is ~10% but the strain in the mucosa and epithelium region is ~25%.

### Emergence and evolution of motility patterns from E5 to E9

We show representative motiligrams of chick gut at stages E5 through E9 in Figs 2 & 3. Videos corresponding to these motiligrams are available as S2–S6 Videos. The total number of guts examined at each stage is: E4 *n* = 5, E5 *n* = 4, E6 *n* = 6, E7 *n* = 8, E8 *n* = 4, E9 *n* = 8.

No motility was present at E4. We detected the first signs of motility at E5. These were restricted to the distal jejunum (next to the umbilicus) and were rhythmic, whole-segment contractions (occurring over a length of ~500μm). At E6, contractile waves were present in the whole gastrointestinal tract (Fig 2) except for the caeca. The contraction was barely detectable (amplitude <1%) in the ileum at this stage. Motion in the caeca was apparent only as from E9 (Fig 3), after they had already grown to a considerable length (~2 mm).

From E6 through E8, contractile wave generation occurred essentially at two sites: the hindgut and the duodenum (or stomach when the latter was not discarded after dissection—see Methods section). The duodenum (or stomach) gave rise to aborally propagating waves. Contractions in the hindgut originated from the middle of this segment, giving rise to a short-distance, aborally propagating wave and a long-distance abanally propagating one. The latter abanal wave propagated through the ileum and umbilicus. It then annihilated in the vicinity of the umbilicus with the aborally propagating waves generated in the duodenum (or stomach). This stereotypical pattern is particularly visible in the motiligrams at E7 and E8 (Figs 2 & 3). At E9, wave nucleation sites were present all along the whole jejunum and ileum, giving rise to more numerous nucleation and annihilation events (Fig 3). Overall, the ileum exhibited the most stable beat, as successive contractile waves propagating in this region were separated by a fairly constant time interval—this is well visible in the E7 motiligram (Fig 2).

### Evolution of the speed, frequency and amplitude of peristalsis waves

**Fig 4:**
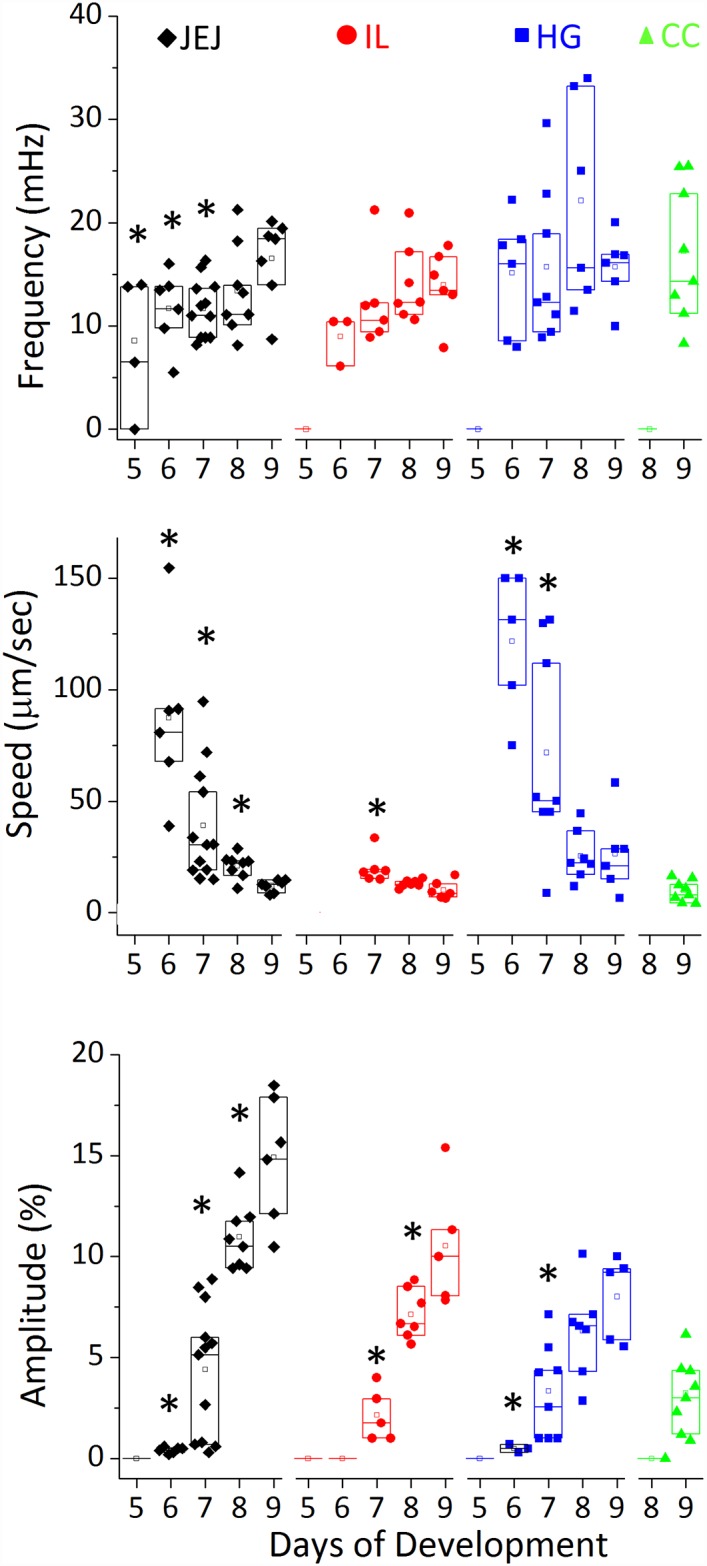
Evolution of peristalsis frequency, wave speed and amplitude as a function of developmental stage. The minimum number of samples analyzed per stage is: E5 n = 4, E6 n = 6, E7 n = 6, E8 n = 8, E9 n = 5. Speed values for E5 guts and E6 ileum could not be determined as rhythmic contractions were present but were very shallow and appeared as bulk, whole-segment constrictions. Propagative constrictions appeared in the caeca only as from E9. A star indicates that the measured value has a statistically significant difference (p<0.05, Mann-Whitney two-tailed test) compared to the value of the same segment at E9.

In Fig 4 we report the evolution of the average frequency, propagation speed and amplitude of the waves in the jejunum, ileum, hindgut and caeca from E5 to E9.

The frequency was near constant and equal to 16±3.5 mHz (~1 cycle per minute) in the hindgut from E5 to E9; it increased from E6 to E9 in the ileum and jejunum from respectively 8.9±1.2 mHz to 14±1.9 mHz (+57%), and 10.8±1.7 mHz to 16.3±2 mHz (+51%). This is consistent with the fact that the number of nucleation sites gradually increased in these gut regions at E8-E9 (Λ-shapes in the motiligrams of Figs 2 & 3). The speed of propagation of the waves decreased (statistically significant) from 87±34 μm/sec in the jejunum and 122±16 μm/sec in the hindgut at E6 to respectively 12±2 μm/sec and 26±9 μm/sec at E9. This is consistent with the fact that the first contractile activity emerging in the gut at E5 consists of whole-segment constrictions, i.e., they propagate too quickly to be detected with the time resolution of our time-lapse videos. Gradually, these constrictions became more localized and started propagating at slower speeds. We also found that contractile wave propagation speeds were greatest in the hindgut and lowest in the ileum. The amplitude of the contractions increased with developmental time in all segments of the gut, reaching up to ~20% at E9. The wave amplitude was in the order A_JEJ_ > A_HG_ ~ A_IL_ > A_CC_.

### The appearance of smooth muscle correlates with the onset of peristalsis

**Fig 5:**
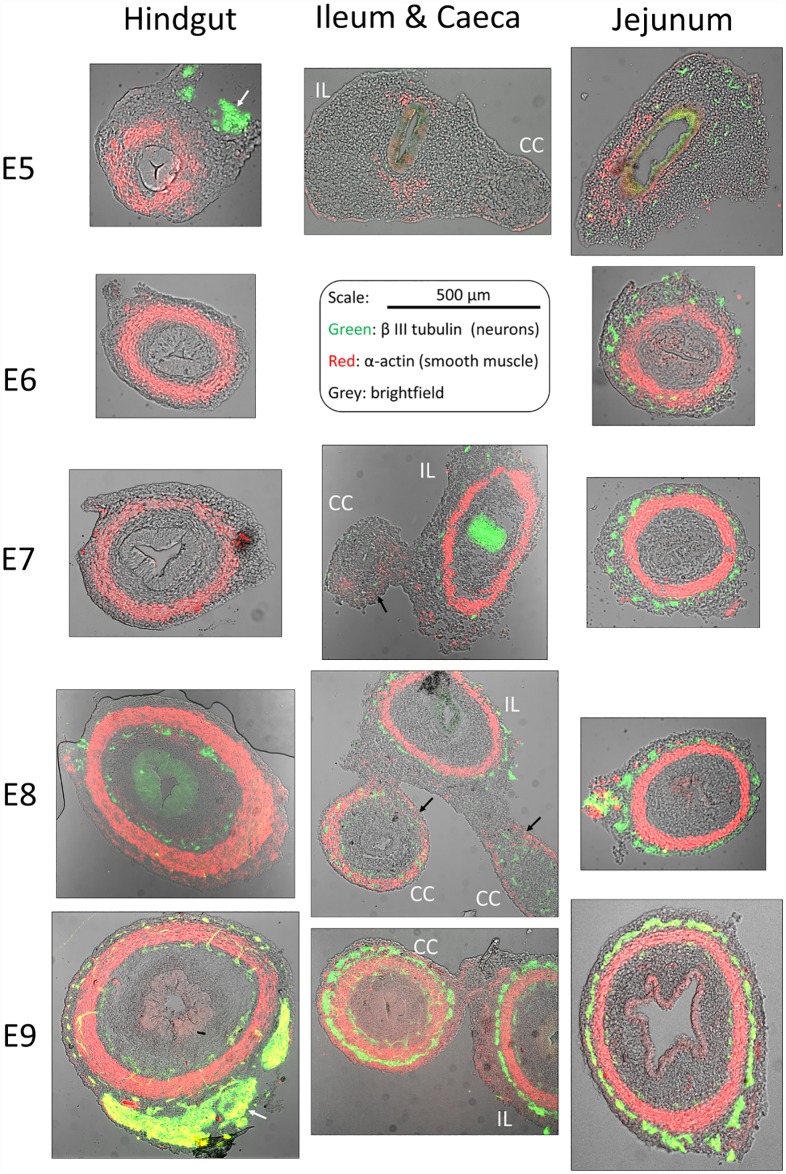
Immunohistochemical staining of neurons (green, β-III tubulin) and smooth muscle (red, α-actin) in the hindgut, jejunum (pre-umbilical midgut) and ileum (IL)—caeca (CC) region from E5 to E9, transverse sections. The brightfield image is overlaid in grey. The scale bar is the same for all sections. The enteric nervous system appears as rings of neurons located around the circular smooth muscle layer; concentrated, non-axisymmetric patches of neurons visible on the periphery of sections E5-HG and E9-HG are the extrinsic innervation (white arrows). The epithelium is visible on the brightfield image, but sometimes stains non-specifically either to Alexa488 (e.g., E7-IL, E8-HG) or to CY3 (e.g., E8-JEJ) secondary antibody.

In order to correlate our kinematic findings to the histology of the developing gut, we performed immunohistochemical staining of smooth muscle (α-actin specific antibody) and enteric neurons (β-III tubulin specific antibody) in thin sections of hindgut, jejunum (pre-umbilical midgut) and caeca from E5 through E9 (Fig 5).

At E5, α-actin positive regions were present in the hindgut and jejunum, but they did not form a closed circular ring. At this stage, the jejunum exhibited sporadic, non-propagative contractility (Fig 2). The caecum and the ileum at E5 lacked any detectable motion and exhibited very faint α-actin staining (Fig 5). We found that the emergence of an α-actin positive, uninterrupted circular ring at E6 in the hindgut and jejunum correlated with the appearance of propagative contractile waves at E6 in those regions. Similarly, α-actin staining in the caecal appendix was weak at E7 and E8 (Fig 5 — IL&CC, black arrows) in comparison to α-actin staining of the ileum at the same rostro-caudal level. In particular, smooth muscle in the caeca did not form a closed circular ring at E7 and E8; no peristalsis motion could be detected in the caeca at those stages. At E9, coinciding with the appearance of peristalsis motion in the caeca, a well-developed band of circular smooth muscle formed and stained with the same intensity as the smooth muscle in the ileum (Fig 5 –IL&CC, E9).

The appearance of β-III tubulin positive cells (neurons) proceeded in a rostro-caudal fashion as described by other investigators [12]. Neurons were present in all (both proximal and distal) hindgut sections by E9. α-actin positive patches and β-III tubulin positive cells appeared at the same time in the jejunum (at E5); they also appeared at the same time in the caeca (at E7). In contrast, in the hindgut, a circular smooth muscle ring was already well developed at E6, 2 days prior to the appearance of neurons at E8. This confirms previous observations by Burns et al. [13]

### Early peristalsis is insensitive to tetrodotoxin but depends on Ca^2+^ channels

**Fig 6:**
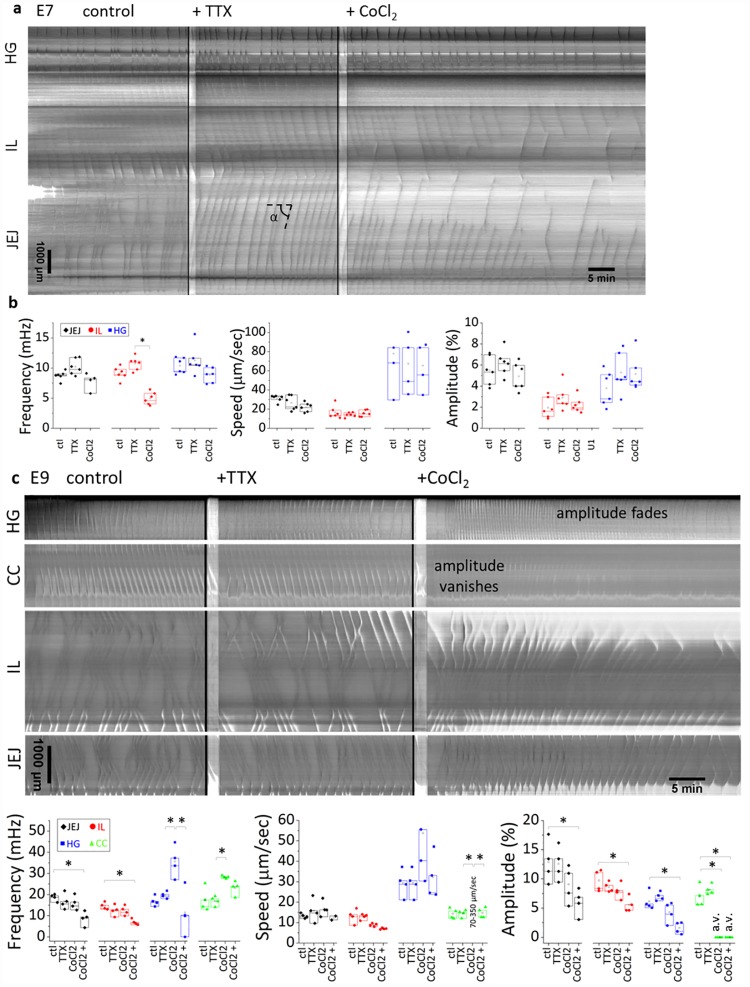
Effect of TTX and CoCl_2_ at E7 (a,b) and E9 (c,d). a,c) Representative motiligrams in control conditions, after the addition of TTX 1 μM and after the addition of CoCl_2_ 1 mM. b,d) Changes in frequency, speed and amplitude after addition of TTX or CoCl_2_. Number of samples: E7 n = 6, E9 n = 4. The speed *v* is related to the tilt angle *α* by *v* = tan *α*. A star indicates a statistically significant difference (p<0.05, Mann-Whitney two-tailed test). In d) we separated the immediate effects of CoCl_2_ from its effect after 20 min application; we also indicate when the amplitude vanishes (“a.v.”) or fades after addition of CoCl_2_. The wave speed in the caecal appendix immediately following CoCl_2_ application was in the range 70–350 μm/sec.

To determine whether neurons are involved in the motility we observed, we used the sodium channel blocker tetrodotoxin (TTX, 1 μM). After thermalization, we first recorded the physiological (control) activity of the guts, and then added TTX to the medium (Fig 6a and 6c). We found that TTX had no significant influence on contractile wave frequency, propagation speed or amplitude at E7 (n = 6, Fig 6a and 6b) and E9 (n = 4, Fig 6c and 6d). Note that in Fig 6, unlike in Figs 1–3, time and space are respectively the x- and y- axis.

To test whether calcium channels are involved in early peristalsis, we imaged motility in calcium- and magnesium-deprived DMEM. Motility was completely abolished in those conditions but was restored after the medium was replaced with DMEM containing Ca^2+^ and Mg^2+^ (E8, n = 3, S7 Video). We further tested the effect of the calcium channel blocker, CoCl_2_ (1 mM). At E7 (n = 6), we found that CoCl_2_ perturbed the propagation of contractile waves, resulting in a premature fading of both aborally and abanally propagating waves respectively in the jejunum and in the ileum (Fig 6a). This resulted in a significant decrease of the wave frequency in these segments (Fig 6b). Wave frequency was not significantly affected in the hindgut; neither were the amplitude or the propagation speed in all segments of the gut at this stage. At E9 (n = 4), the most striking effect of CoCl_2_ was the immediate extinction of contractile wave amplitude in the caeca (Fig 6c, S8 Video); faint, high-velocity traveling “shadows” could still be detected, but they were not accompanied by contractions of the walls of the caeca. The hindgut reacted by displaying at first high frequency (about ~2 times the frequency in control conditions) peristalsis waves. This excited state was followed about ~20 min later by an almost complete extinction of peristalsis amplitude and frequency in this segment (Fig 6c and 6d). The amplitude and frequency in the ileum and jejunum were not perturbed immediately after CoCl_2_ application but significantly decreased after 20min application (Fig 6d). We also observed that application of CoCl_2_ at E9 induced a gradual increase in gut diameter (+22.5±5.1%, after 1H application, *n* = 4, S8 Video), accompanied by a reduction in length of all gut segments (-23±3.1%, after 1H application, *n* = 4, S8 Video). This increase in diameter could be due to relaxation of circular smooth muscle tone after calcium channel blocking. This contractile effect was not observed at E7.

### The development of motility of cultured guts parallels that seen in-vivo

**Fig 7:**
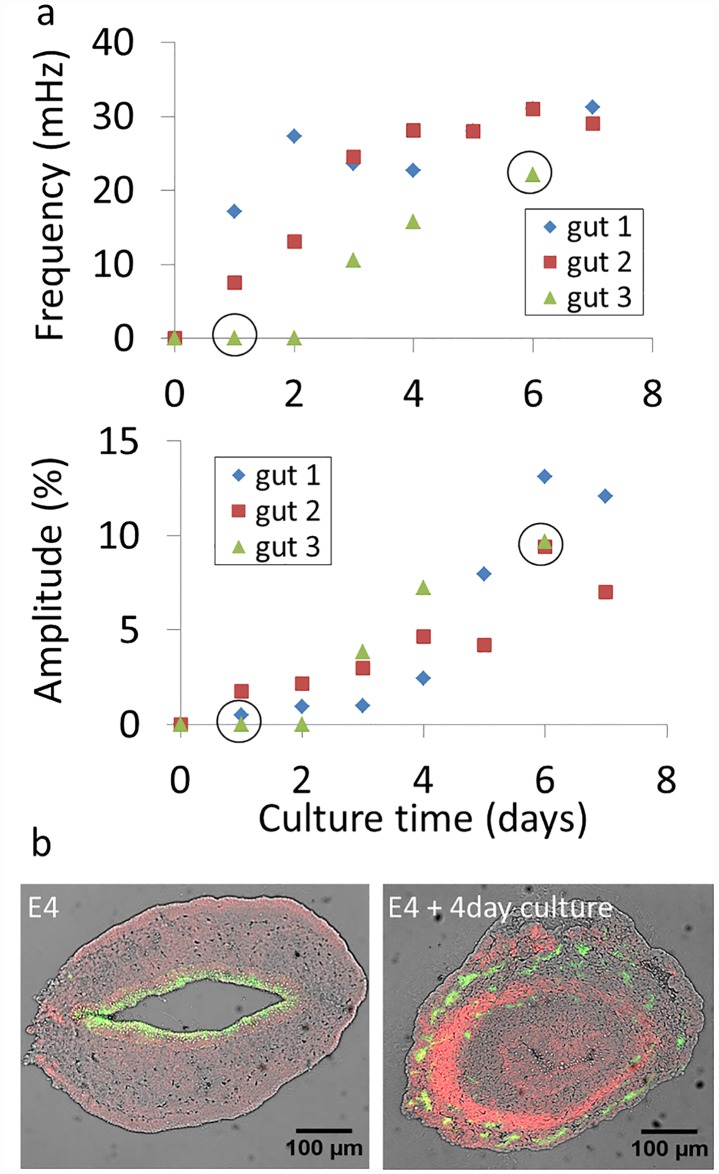
a) Evolution of jejunal contractile wave frequency and amplitude of E4 guts (n = 3) kept up to 7 days in culture. Circled data points indicate that the full time-lapse video corresponding to this condition is available as S9 & S10 Videos. b) Immunohistochemical staining of neurons (green, β-III tubulin) and smooth muscle (red, α-actin) in native E4 midgut (left) and E4 midgut kept 4 days in culture (right). The brightfield image is overlaid in grey. For the native E4 midgut, the epithelium stains weakly and non-specifically to Alexa488.

Like cells and many other embryonic organs [14], the GI tract can be dissected from the embryo and kept alive in *ex-vivo* organ culture [15,16]. We wondered whether the physiological evolution of peristalsis we observed (Figs 2–4) would also take place *ex-vivo*, in organ culture. We dissected embryonic guts at stage E4, when no motility was present, and monitored the evolution of jejunal contractile wave frequency and amplitude every day for up to seven days in culture. The results are shown in Fig 7.

For all guts considered, the frequency and contraction amplitude increased with culture time, paralleling the physiological increase in amplitude and frequency we presented above. During the first two days of culture, a regular beat could be observed but it did not propagate; it started propagating as from the third day of culture. We further found that an α-actin positive smooth muscle ring appeared after 4-day culture (*n* = 2, Fig 7b, right); no α-actin was present at E4 (*n* = 2, Fig 7b, left). Although motility developed in culture, the guts did not lengthen (as already observed by Hearn et al. [16]). This resulted in miniature, forcefully pumping organoids, some examples of which are shown in S9 & S10 Videos.

### Motility emerges in cultured aganglionic hindguts

Ruggiero et al. found TTX-resistant Na^+^ currents in duodenum myenteric neurons of adult rat and guinea pig [17]. To unambiguously determine the involvement of neurons in early peristalsis, we therefore performed another experiment in which aganglionic E5 hindguts (segment comprised between the cloaca and the caecal appendix-ileum junction) were dissected out and cultured for 3 days in DMEM. No motility was present in the hindgut at E5 (Figs 2 & 4). After 3 day culture, all samples (*n* = 4/4) exhibited propagating contractile waves in the hindgut (S11 Video, left). The mean amplitude, frequency and speed (*n = 4* ± standard deviation) were respectively 4.3±1.6%, 21.2±5 mHz and 71±65 μm/sec. Post-culture immunohistochemistry (*n = 4)* of these hindguts showed the presence of a well differentiated smooth muscle actin ring and confirmed that these cultured hindguts lacked enteric neurons (S11 Video, right, staining of the same sample after the video was recorded). This unambiguously demonstrates that neurons are not required for the emergence of early motility.

## Discussion

We have found that rhythmic contractility first appears at E5 in the chicken jejunum; by E6 all regions of the gut except the caeca undergo peristalsis-like motion; motility appears in the caeca only as from E9. From E6 to E9, the general pattern of contractility is dominated by two contractile wave sources, the hindgut and the duodenum (or stomach). These two sources emit counter-propagating contractile waves which meet and annihilate in the vicinity of the umbilicus. The dynamic pattern of contractility (motiligram) becomes more complex at E9 as wave nucleation sites emerge all along the midgut. Development of peristaltic activity from E6 to E9 is characterized by a gradual increase in contraction amplitude, a decrease in propagation speed, and a 30–50% increase in contraction frequency in the midgut. The strain due to peristaltic waves is concentrated in the epithelium and mucosa. The emergence of peristaltic activity in the hindgut, midgut and caeca coincides with the appearance of a well-defined and uninterrupted, α-actin positive band of circular smooth muscle in those regions. We have shown that the contractile activity does not require the input of neurons, as the dynamical contractility patterns is unaffected by a potent sodium channel blocker (tetrodotoxin, at E7 and E9), and motility was seen to emerge in cultured hindguts lacking enteric neurons. It however relies crucially on calcium channels, as calcium deprivation completely inhibits motility, and cobalt chloride, a calcium channel blocker, leads to disruptions of motility at E7 and E9. We have finally shown that motility, unlike overall organ size, develops in a simple organ culture setup.

Enteric neural crest cells (ENCCs) invade the embryonic chick midgut from E4 to E6 and the hindgut from E6 to E8 [12]. Differentiation to neurons and glia takes place in a rostro-caudal wave that is offset by about ~1 day (E5 to E9) with respect to ENCC migration [18]. Our results confirm that in the chicken, the emergence of a smooth muscle ring in the hindgut (E6) precedes the colonization of this region of the gut by neural crest cells [13]. We additionally show that rhythmic contractile activity takes place in this region at E6 in the absence of neurons, and that cultured hindguts lacking enteric neurons are motile (S11 Video). Harrison [19] demonstrated more than a century ago that muscle differentiation could proceed independently of innervation. We note however that Faure et al. [20] found that development of smooth muscle in the embryonic chick stomach was impeded when neural crest cell number was reduced by somite 3–6 ablation at HH9-HH11. In the mouse embryo, emergence of motility in the hindgut [7] takes place around the same time as terminal hindgut invasion by ENCCs (E14.5); Carey [21] mentioned that in the descending pig colon, smooth muscle differentiation had taken place before Auerbach’s plexus could be detected but we could not find any reference or proof of this assertion. In the zebrafish, enteric neurons are detected throughout the whole gut at 3dpf, 1 day before the onset of motility [22]. In the human gut [23], appearance of α-actin immunoreactivity (weeks 7–9) occurs after ENCC invasion (weeks 4–7) and coincides with the segregation of enteric neurons in myenteric and submucosal plexus; no data pertaining to early (<15 weeks) motility is available for human embryos, because of the lack of suitable experimental techniques to monitor this motion during pregnancy.

The fact that TTX does not affect gut motility at E7 and E9, although neurons are well differentiated respectively in the midgut and in the whole gut by that time, indicated that neurons do not participate in gut peristalsis patterns at these stages. This extends results by Holmberg et al. [8] and by Roberts et al. [7] showing that neurons were not involved in gut motility in respectively the zebrafish at 4 dpf and the embryonic mice from E13.5 to E19.5 (*i*.*e*., two days before birth), although a well-developed enteric nervous system is present in these species at these developmental stages. Roberts et al. additionally showed that ICC-depleted mutant mice exhibited the same motility as wildtype mice at E16.5, indicating that motility from E13.5 to E16.5 is purely myogenic. In the chicken, Le Douarin et al. [24] have traced back the appearance of ICCs in the gut to between E7 (faint c-kit in-situ hybridization staining) and E9 (marked dot like staining in the region of Auerbach’s plexus). As we have observed motility starting at E5-E6, our results are in line with the idea that the first motility patterns (from E5 to E7 at least) are independent of ICCs and neurons, *i*.*e*., they are purely myogenic. The presence of ICCs at E9 could be responsible for the increased number of contractile wave nucleation sites we observed throughout the midgut at this stage (Fig 3). We unfortunately do not have at our disposal a simple way of turning down ICC activity in the chicken embryo to determine their contribution to motility once these cells have differentiated (E7-E9). Blocking calcium channels in mice at E16.5 and E18.5 using CoCl_2_ completely abolished myogenic peristalsis [7]. We found a less pronounced effect in chickens, as CoCl_2_ only abolished peristalsis in the caeca (immediate) and hindgut (after 20 min) at E9; it perturbed but did not abolish motility in the midgut at E9 and E7 (Fig 6).

The orders of magnitude of peristaltic wave frequency, amplitude and propagation speed in the chicken from E5 to E9 are respectively 10–20 mHz, 0–20% and 10–150 μm/sec (Fig 4). These values are similar to those found by Holmberg et al. [22] for 3 to 7 dpf zebrafish embryos: 10–50 mHz, 10–25 μm/sec. In line with our findings (Fig 4), these investigators observed that the propagation velocity decreased with increasing developmental time from 4 dpf to 7 dpf [8]. This decrease in velocity with increasing age could be due to the fact that the amplitude of the wave and the mass of tissue to be displaced increases with age; it could also be due to changes in inter-cellular (e.g., gap junctions) signal conduction velocity. Roberts et al. [7] measured the following characteristics for “ripples” (propagating myogenic contractions) in E16.5 mouse embryos: 40 mHz, 24%, 220 μm/sec in the duodenum and 16 mHz, 20% and 40 μm/sec in the colon. The speed of propagation of contractions in mouse embryos is noticeably higher than those found here or for zebrafish embryos. We note that peristalsis frequency and propagation speed are affected by the precise composition of the physiological medium used for monitoring motility and in particular by the amount of divalent ions [25]; this should be taken into account when comparing speeds obtained from different studies.

We have found that from E6 to E8, peristaltic waves propagated aborally and abanally from the stomach and from the hindgut; these counter-propagating waves annihilated at the umbilicus, giving rise to an overall rather counterintuitive motiligram, as one would expect the waves to travel on average in an aboral direction; of course, this pattern may simply be witness to the fact that peristalsis is not yet adapted for bolus transport at these early stages. Also, we cannot exclude the possibility that the *in-ovo* direction of propagation of peristalsis waves may be affected by electric potential differences in the embryo that differ from the ones present after dissection and isolation of the organ. Other investigators studied isolated gut segments and could not draw conclusions as to the overall propagation pattern. Dissection of embryonic chick guts at E10 revealed the presence of conspicuous green bile salt down to the level of the distal ileum, indicating that peristalsis was effective at transporting fluid present in the lumen in an aboral direction at least as from E10. Similar observations were made on wildtype and *Ret*-null embryonic mice guts at E17.5 [26].

Previous investigators have shown that cells in cultured embryonic guts continue migrating [15] and differentiating [16]. Here we show that peristalsis develops in a simple *ex-vivo* organ culture system. While muscular contractions became more and more powerful, the overall size of the cultured organs did not increase: this shows that growth and differentiation of this embryonic organ follow two independent routes, i.e., function (peristalsis) does not require form (gut size and shape). Conversely, we see that the caecal appendix can grow to a considerable length (up to ~2mm at E9) without exhibiting any contractile wave activity in the period from E6 to E8. We therefore also see that for the early caecal appendix, form (growth) does not require function (peristalsis).

In conclusion, we have presented an extensive description of the emergence of motility in the embryonic avian gut from E5 (first signs of motility) to E9. We have integrated the development of motility in the chronology of other major developmental events (ENCC invasion, appearance of ICC and smooth muscle) in the chicken gut, and compared this enhanced chronology to that of other vertebrate animals (mouse, zebrafish, human). We have shown that, just as for the mouse and zebrafish, early motility does not require neuronal activity (E5 to E9) or ICCs (E5 to E7), but is critically dependent on smooth muscle and ionic calcium fluxes. We have finally shown that the emergence of peristalsis can be studied in an *ex-vivo* culture system; in such conditions, peristalsis was found to develop independently from organ growth. Future investigations will be aimed at understanding the essential physical underpinnings and the physiological role of early myogenic propagative contractions in the developing embryonic gut.

## Methods

### Specimen preparation

Fertilized chicken eggs were purchased from EARL Morizeau (Chartres, France). The eggs were incubated at 37.5°C in a humid atmosphere for 5 to 10 days. The gastrointestinal tract was dissected out from hindgut to duodenum. The mesentery was carefully removed with tweezers so that the gut could be straightened out for imaging. The experiments were conducted under French law article R214-87 modified by the Décret n°2013–118 to comply to European Union regulations. The approval by an ethics committee is not required for research conducted on chicken embryos within the first two thirds of their development.

### Motility monitoring setup

E7 to E9 guts were placed on Anodisc membrane discs (Whatman, pore size = 0.2 μm, Ø = 47 mm) resting on the edge of a tissue culture dish (Ø = 40 mm) filled with DMEM GlutaMAX-I (Life Technologies, with 4.5 g/L D-glucose and sodium pyruvate, [Ca^2+^] = 1.8 mM, [Mg^2+^] = 0.8 mM) complemented with 25 mM HEPES. Up to three guts were placed on the same membrane. The level of DMEM was adjusted so that the guts were in contact with DMEM from below by capillarity, and exposed to atmospheric oxygen from on top. The liquid meniscus pressed the guts against the substrate, providing a convenient way of preventing bulk movements of the samples. The tissue culture dish + Anodisc + guts were next placed in a closed humidified Petri dish and thermalized at 37.5°C. E5 and E6 guts were very soft and considerably deformed by the meniscus pressure, resulting in altered motility. We therefore resorted to a different protocol to image the motility of these guts. We pinned up to four E5-E6 guts to a Sylgard-coated cylindrical cell and subjected them to a constant flow of DMEM kept at a constant temperature of 37.5°C and bubbled with carbogen (95% O_2_/5% CO_2_). We tested both the flow and the Anodisc protocol for E7 guts and found no significant difference of motility between these two protocols.

We thermalized the samples for 30 min before recording; time-lapse imaging was performed for up to 3 hours at frequencies ranging from 0.25Hz to 1Hz with a Leica Z16 APO binocular equipped with a 1600x1200 px B&W CCD camera. Chemicals used were: CoCl_2_ (Sigma-Aldrich, 1 mM), tetrodotoxin (Abcam, 1 μM), Ca^2+^- and Mg^2+^-free DMEM (ThermoFisher Scientific). Motility was analyzed using ImageJ software as described in the results section. All videos in the Supporting Information part are in AVI format; frame rate of all videos except S8 Video 8: 4 sec/frame; frame rate of S8 Video. 8 sec/frame. All analysis were performed on HD videos with a 2-times higher spatial resolution and 2- to 4-times higher temporal resolution.

### Embryonic gut culture

To study the development of motility in culture, the dissected and demesenterized guts (hindgut to stomach) were placed at the bottom of individual tissue culture dishes (Ø = 40 mm) filled with a 1 mm deep layer of DMEM supplemented with 1% penicillin-streptomycin and cultured in a humidified incubator (Thermos) at 37.5°C in a 5%CO_2_ / 95% air atmosphere. Medium changes were performed every other day. The motility of the cultured guts was imaged every day for ~20 min directly in the tissue culture dish, at 37.5°C.

### Immunohistochemistry

Guts were fixed for 1H in a 4% PFA in PBS solution, washed in PBS, then let overnight in 30% sucrose in water solutions, and embedded the next day in OCT compound (VWR). 14 μm slices were cut at -20°C with a cryotome (Leica) and deposited on Thermofrost glass slides (VWR). After rehydration and blocking of the slides for 15 min in a 1% BSA in PBS solution, the slides were then incubate overnight in an anti-α smooth muscle actin antibody (Abcam, ref5694, dilution 1:2000) and anti βIII-tubulin antibody (Abcam, ref14545, dilution 1:1000) solution composed of 0.15% BSA in PBS; the following day, after washing, complementary fluorescent secondary antibodies (CY3 and Alexa488, dilution 1:400 in PBS) were applied for 2H. The slides were washed, sealed with a coverslip and immediately imaged with an inverted epifluorescence microscope (Leica).

### Statistical analysis

All pairwise statistical analysis were performed using a two-tailed Mann-Whitney test. Differences were considered statistically meaningful at p <0.05 (indicated by a star in Figs 4 & 6). The box plots in Figs 4 & 6 represent the median, upper and lower interquartile; the mean is represented by an empty square.

## Supporting information

S1 Video High magnification view of peristalsis in E8 jejunum (cf Fig 1). (AVI)

S2 Video Activity of E5 gut (cf Fig 2). (AVI)

S3 Video Activity of E6 gut (cf Fig 2). (AVI)

S4 Video Activity of E7 gut (cf Fig 2). (AVI)

S5 Video Activity of E8 gut (cf Fig 3). (AVI)

S6 Video Activity of E9 gut (cf Fig 3). (AVI)

S7 Video Comparison of motility in Ca^2+^-deprived medium and after substitution with normal medium (Ca^2+^ 1.8 mM), E8 gut. (AVI)

S8 Video Effect of TTX and CoCl_2_ on an E9 gut (cf Fig 6). (AVI)

S9 Video Activity of E4 gut cultured for 1 day (cf Fig 7). (AVI)

S10 Video Activity of E4 gut cultured for 6 days (cf Fig 7). (AVI)

S11 Video Left panel: activity of E5 aganglionic hindgut cultured for 3 days, the hindgut is on the right. Right panel: immunohistochemical staining of enteric neurons (Tuj, green) and smooth muscle (α-actin, red) of the fixed hindgut after the video was recorded. The epithelium exhibits weak non-specific staining to Alexa488.
(AVI)
